# 3D Bioprinted Natural Hydrogels: Rheological Characterization, Cytotoxicity, and Printability Assessment of a Polysaccharide-Based Bioink

**DOI:** 10.1021/acsomega.5c09668

**Published:** 2026-01-05

**Authors:** David Picado-Tejero, Laura Mendoza-Cerezo, Jesús M. Rodríguez-Rego, Antonio Macías-García, Alfonso C. Marcos-Romero

**Affiliations:** † Departamento de Expresión Gráfica, Escuela de Ingenierías Industriales, 16759 Universidad de Extremadura, Avenida de Elvas, s/n, 06006 Badajoz, Spain; ‡ Departamento de Bioquímica, Facultad de Ciencias, Universidad de Extremadura, Avenida de Elvas, s/n, 06006 Badajoz, Spain; § Departamento de Ingeniería Mecánica, Energética y de los Materiales, Universidad de Extremadura, Escuela de Ingenierías Industriales, Avenida de Elvas, s/n, 06006 Badajoz, Spain

## Abstract

The creation of natural bioinks suitable for three-dimensional (3D) bioprinting remains a significant challenge in developing functional and biocompatible materials for tissue engineering. In this work, a novel bioink formulation was designed using food-grade polysaccharides, κ-carrageenan (KC), tragacanth gum (TG), and konjac glucomannan (KG), and thoroughly evaluated. Each component was tested at varying concentrations through rheological analysis, in vitro cytotoxicity assays (MTS test with HEK293T cells), and printability assessments using a commercial bioprinter. Optimal concentration ranges were identified as ≥2% for KC, ≥1.5% for TG, and 1.5–2% for KG, which were then combined into two candidate hydrogel formulations (A and B). Both exhibited viscoelastic behavior and pseudoplastic flow characteristics. Formulation B (2% KG) demonstrated greater structural rigidity (*G*′ ≈ 40 kPa) and excellent print fidelity (>84%) under multiple extrusion conditions, while formulation A (1.5% KG), though mechanically less robust, showed superior biocompatibility, achieving 86.5% cell viability after 24 h and 82.1% after 48 h. Overall, the study underscores the promise of food-derived polysaccharides as sustainable and customizable bioink components, with potential applications in engineered tissue scaffolds, in vitro models, and biocompatible 3D-printed systems.

## Graphical abstract

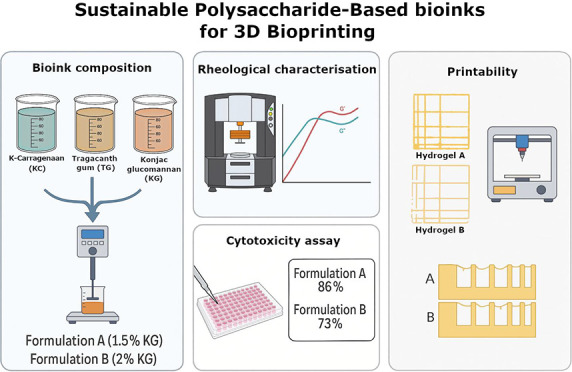

## 1 Introduction

Three-dimensional (3D) bioprinting has emerged as a key technology in bioengineering, enabling the creation of biomimetic structures based on patient-specific data, thus allowing for a truly personalized approach.^1,2^ Despite substantial progress in the field of 3D bioprinting, the development of hydrogel-based bioinks that simultaneously ensure mechanical integrity, print fidelity, and cytocompatibility remains a major challenge. These materials must withstand the mechanical and thermal stresses associated with extrusion while displaying viscoelastic properties that ensure structural stability and shape retention without compromising cell viability.^3–5^

Current bioinks, whether synthetic or natural, often exhibit trade-offs between rheological performance and biological functionality. Therefore, optimizing the rheological properties of formulations is essential to ensure both structural fidelity and the biological functionality of the printed construct.^6^

Synthetic hydrogels, while mechanically robust and tunable, frequently involve toxic cross-linkers or nonbiodegradable components that limit clinical translation.^7^ Conversely, natural polymers typically offer excellent biocompatibility but lack the structural strength required to preserve the printed geometry, especially under physiological conditions.^8^ As an intermediate solution, hybrid hydrogels combine natural and synthetic polymers to integrate the advantages of both types.^9,10^ However, challenges such as phase incompatibility, cytotoxic cross-linking, and reduced bioactivity continue to limit the reproducibility and long-term applicability of hybrid hydrogels in bioprinting,^11,12^ often leading to the inheritance of weaknesses from both constituent components. Specifically, for nanomaterial-based hybrid hydrogels, additional concerns include the fate of the nanomaterials in biological environments and the lack of regulatory approval, which must be addressed prior to clinical translation.^13^

Furthermore, commercial bioinks are limited in variety, costly, compositionally undefined, and often derived from nonrenewable sources, raising concerns regarding reproducibility, scalability, and sustainability.^14^ These limitations have prompted increasing interest in the design of new, low-cost, and environmentally sustainable hydrogels derived from natural polysaccharides with well-characterized composition and biocompatibility profiles.

Developing new hydrogels from well-characterized, food-grade polysaccharides represents a sustainable and innovative approach for next-generation bioinks.^15^ These materials offer inherent biocompatibility, tunable viscoelastic properties, and cost-effectiveness while minimizing environmental and regulatory concerns.

κ-Carrageenan (KC), tragacanth gum (TG), and konjac glucomannan (KG) are particularly promising candidates due to their complementary physicochemical, rheological, and biological features. KC, food additive E-407, is a sulfated polysaccharide derived from red algae of the order *Rhodophyceae* that forms thermo-reversible gels in the presence of potassium ions. Its repetitive galactose-based structure and approximately 20% sulfate group content make it valuable in food and biotechnological applications.^9,16–18^ TG, food additive E-413, is an anionic, branched polysaccharide obtained from species of the *Astragalus* genus. It forms hydrogels through ionic interactions or hydrogen bonding and has demonstrated biocompatibility, immunomodulatory effects, and applications in dermal devices.^19–22^ Finally, KG, food additive E-425ii, is a water-soluble heteropolysaccharide with neutral pH, extracted from the *Amorphophallus konjac* plant. It can stabilize proteins and exhibits prebiotic, anti-inflammatory, and antitumor effects, reinforcing its potential as a functional component in bioinks.^23–26^

This study introduces a multicomponent hydrogel system composed of three food-grade polysaccharides as a versatile and sustainable alternative for extrusion-based 3D bioprinting. By combining the thermos-reversible gelation of KC with the stabilizing and bioactive effects of TG and KG, the proposed KC–TG–KG system seeks to overcome the persistent trade-off between structural fidelity and cytocompatibility. The systematic assessment of its rheological behavior, cytotoxicity, and printability provides a reproducible framework for designing bioinks with tunable mechanical and biological properties. This approach addresses a recognized gap in the field and supports the development of safer, more effective, and sustainable bioinks for tissue engineering and regenerative medicine.

## 2 Materials and Methods

### 2.1 Materials and Reagents

Three food-grade natural polysaccharides with recognized biocompatibility were used: κ-carrageenan (KC), tragacanth gum (TG), and konjac glucomannan (KG). KC (ref 22048–100G-F, purity >99%^27^) and TG (ref G1128–100G, purity not provided by the supplier) were purchased from Sigma-Aldrich (St. Louis, MO, USA). KG, purity ≥ 90%, was supplied by Shaanxi Bohong Health Industry Co., Ltd. (Shaanxi, China).

Solutions were prepared using PBS tablets from Sigma-Aldrich (0.01 M Phosphate Buffer, 0027 M Potassium Chloride, 0.137 M Sodium Chloride) in deionized water, with the pH adjusted to 7.4.

For cytotoxicity assays, the human cell line HEK293T was used, provided by the Research Support Service of the University of Extremadura. Cell culture was carried out in DMEM medium (Corning) supplemented with 10% fetal bovine serum (FBS), under controlled conditions at 37 °C and 5% CO_2_. The medium’s pH was monitored periodically using ECENSE test strips. Cytotoxicity was assessed using the CellTiter 96 AQueous One Solution Cell Proliferation Assay (Promega), which is based on the reduction of MTS in the presence of phenazine methosulfate (PMS). Measurements were performed with a HiPo MPP-96 microplate reader (Biosan).

Rheological characterization was conducted using a Kinexus Prime Pro+ rotational rheometer (Netzsch, Germany). For printability analysis, a BIO X bioprinter (CELLINK) and AutoCAD 2023 software (Autodesk Inc.) were used.

### 2.2 Characterization of Polysaccharides

#### 2.2.1 Kappa-Carrageenan

Carrageenans are linear, anionic polymeric chains with molecular weights ranging from 453 to 652 kDa. In the case of κ-carrageenan, the structure is characterized by the presence of sulfate ester groups, consisting of alternating units of [(1 → 3)-β-d-galactopyranose-4-sulfate-(1 → 4)-3,6-anhydro-α-d-galactopyranose]*_n_*,^16^ with a sulfate content of approximately 20% (w/w)^28^ (Figure 1). The degree of sulfation is directly associated with enhanced gel strength and gelling temperature, κ-carrageenan being the strongest among the carrageenans.^29^ Regarding its electrostatic interactions, in the presence of cations, κ-carrageenan forms helical structures that lead to rigid gels, particularly in the presence of K^+^ ions.^30^ In the absence of cations, gelation with lower rigidity may also occur through cooling-induced cross-linking.

**Figure 1:**
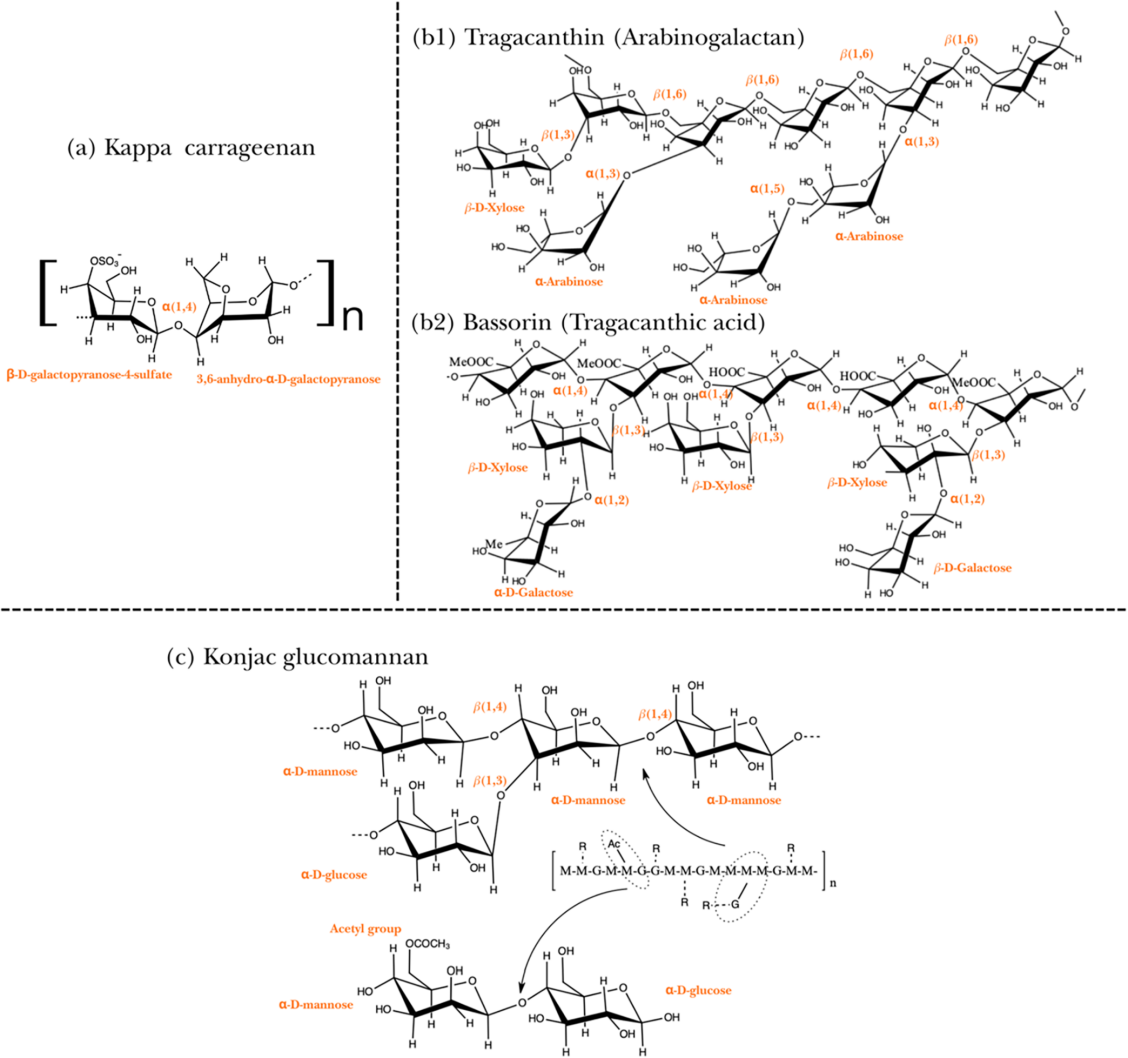
Chemical structures of the utilized polysaccharides. (a) Repeating disaccharide unit of KC composed of d-galactose-4-sulfate and 3,6-anhydro-d-galactose. (b) Principal fractions of TG: (b1) Tragacanthin (water-soluble fraction) and (b2) Bassorin (swelling fraction), highlighting the presence of carboxylic acid and methyl ester groups on the backbone. (c) Structural representation of KG (repeated units) depicting the random distribution of β-1,4-linked d-mannose (M) and d-glucose (G) units, along with acetyl groups and branching points (R). Developed by the authors.

#### 2.2.2 Tragacanth Gum

Tragacanth gum (TG) is a branched, heterogeneous, negatively charged polysaccharide capable of undergoing ionic cross-linking in the presence of cations. It exhibits a slightly acidic character and has an approximate molecular weight (MW) of up to 850 kDa.^31^ TG consists of two main fractions, which vary depending on the botanical source: *tragacanthin* (also referred to as arabinogalactan, water-soluble, 30–40% w/w) and *tragacanthic acid* (also referred to as bassorin, water-swellable, 60–70% w/w).^32^ These two fractions are not chemically bound and typically occur in ratios ranging from 1:2 to 1:4.^33^

More specifically, tragacanthin is composed of l-arabinose, l-fucose, d-mannose, d-glucose, d-galactose, and d-xylose residues, whereas bassorin contains l-fucose, d-xylose, d-galacturonic acid, d-galactose, and l-rhamnose^34^ (Figure 1). The ratio of tragacanthin to bassorin varies across *Astragalus* species.^31^ According to Sigma-Aldrich, TG extracted from *Astragalus gummifer* displays a composition of approximately 40:60 (tragacanthin:bassorin).^35^

#### 2.2.3 Konjac Glucomannan

According to the supplier, Konjac glucomannan (KGM) has a purity ranging from 95–99%. Structurally, it is a branched polysaccharide with a molecular weight ranging between 200 and 2000 kDa.^36^ Its main backbone consists predominantly of randomly distributed β-1,4-linked α-d-mannose and α-d-glucose residues in a ratio of approximately 1.6:1, along with β-1,3-glycosidic linkages in a ratio of about 1:1.5 (β-1,3:β-1,4).^37,38^ These β-1,3-glycosidic linkages occur at the C-3 position of mannose and glucose residues in the backbone, conferring additional flexibility to the chain^39^ (Figure 1). Moreover, short side branches composed of 3–4 monosaccharide units of glucose and mannose are present, with an average of three branches for every 32 residues of the main chain.

KGM is capable of forming hydrogen bonds and van der Waals interactions with water molecules in aqueous solution due to its high content of hydroxyl and carbonyl groups.^37^ Random acetylation occurs at the C-6 position of glucose units, with acetyl groups found approximately once every 9–20 sugar residues.^40^ These acetyl groups contribute to maintaining the primary structural stability of KGM.^39^ They also enhance polysaccharide solubility in water by preventing excessive interchain hydrogen bonding and aggregation, while further being implicated in the formation of the irregular helical conformation observed in KGM.^36^

### 2.3 Preparation of Base Hydrogels

Stock solutions of KC, TG, and KG were prepared by dissolving each polysaccharide individually in PBS. Concentrations ranged from 1 to 3% (w/v) in 0.5% increments, and an additional 4% (w/v) TG solution was included due to its distinct rheological behavior at higher concentrations.

The solutions were homogenized by magnetic stirring at 80 °C until complete dissolution. If the pH deviated from the physiological value, it was adjusted using sterile PBS. Each formulation was labeled according to the polysaccharide type and concentration, as detailed in Table 1.

**Table 1:** Preparation of Stock Hydrogel Solutions Based on κ-Carrageenan (KC), Tragacanth Gum (TG), and Konjac Glucomannan (KG)^a^

stock solutions of hydrogels K-carrageenan (KC)		stock solutions of hydrogels tragacanth gum (TG)		stock solutions of hydrogels konjac glucomannan (KG)	
tag	concentration %(w/v)	tag	concentration %(w/v)	tag	concentration %(w/v)
1KC	1	1TG	1	1KG	1
1.5KC	1.5	1.5TG	1.5	1.5KG	1.5
2KC	2	2TG	2	2KG	2
2.5KC	2.5	2.5TG	2.5	2.5KG	2.5
3KC	3	3TG	3	3KG	3

a Each formulation is designated according to its specific concentration and the type of polymer used.

Subsequently, the solutions were cooled gradually to room temperature (20–25 °C) in two stages: from 80 to 60 °C at 1 °C min^–1^ under continuous stirring, and from 60 to 25 °C at 0.2 °C min^–1^ without agitation. This two-stage cooling protocol ensures gradual helix formation and network stabilization. The mixtures were then equilibrated at room temperature for 25 min and stored at 4 °C, protected from light, until further use.

### 2.4 Rheological Characterization

Rheological characterization was performed on both individual stock solutions (KC, TG, KG) and selected composite formulations, to evaluate their viscoelastic behavior and determine their suitability for bioprinting applications.

Tests were carried out using a Kinexus Prime Pro+ rotational rheometer (Netzsch, Germany), equipped with a parallel plate geometry (40 mm diameter, 0.5 mm gap), at a controlled temperature of 25 ± 0.5 °C. The following viscoelastic parameters were determined: storage modulus (*G*′), associated with the elastic component, and loss modulus (*G*″), representative of viscous behavior.

Apparent viscosity was also assessed through shear rate sweeps (0.1–100 s^–1^), and thermal sweep tests (20–60 °C) were performed to detect temperature-sensitive structural transitions.

Rheological profiles revealed the pseudoplastic behavior characteristic of the systems studied, as well as potential synergistic or antagonistic interactions in the blends. These data were essential in selecting formulations with higher structural stability, controlled extrusion capability, and postdeposition recovery, key attributes to ensure reproducible bioprinting with high geometric fidelity.

Finally, to evaluate the ability of the hydrogel formulations to recover their internal structure after the mechanical stresses associated with extrusion, a three-interval thixotropy test (3ITT) was performed at 37 °C. This rheological test allows quantifying the structural recovery capacity of shear-thinning materials, which is a critical property for bioinks intended for extrusion-based bioprinting. The assay consisted of three consecutive shear-rate steps: an initial low-shear interval (0.1 s^–1^ for 5 s) to establish the baseline viscosity, followed by a high-shear interval (150 s^–1^ for 60 s) simulating the mechanical conditions experienced during printing, and a final low-shear interval identical to the first step to monitor the structural rebuilding process. The recovery of viscosity during the last interval was used as an indicator of the formulation’s ability to regain its network structure after shear-induced disruption.

### 2.5 Cytotoxicity Assay

Biocompatibility of the formulations was evaluated through cytotoxicity assays, performed on both individual stock solutions (KC, TG, and KG) and final composite formulations. Hydrogels were sterilized by autoclaving (121 °C, 1 atm).

Assays were conducted using human HEK293T cells, cultured in Dulbecco’s Modified Eagle Medium (DMEM, Corning), supplemented with 10% fetal bovine serum (FBS). Culture conditions included incubation at 37 °C with 5% CO_2_. Sterile PBS (Sigma-Aldrich) was used for washing steps, and the medium pH was periodically monitored with ECENSE test strips.

Cytotoxicity analysis was performed after 24 and 48 h of exposure to the hydrogels, using the MTS colorimetric assay (CellTiter 96, Promega). The resulting color intensity, proportional to the number of viable cells, was quantified via spectrophotometry using a HiPo MPP-96 microplate reader.

Each experimental condition was assessed in six independent replicates (*n* = 6). Results were expressed as the percentage of relative cell viability compared to the control group (set at 100%) and presented as mean ± relative standard deviation (RSD). Statistical analysis was carried out using one-way ANOVA followed by a Student’s *t* test as post hoc analysis. Significance was considered at *p* < 0.05 (*), *p* < 0.01 (**) and *p* < 0.001 (***).

### 2.6 Printability Assay

The ability of the selected formulations to generate stable 3D structures was analyzed following the protocol described by Rodríguez-Rego et al.,^43^ using a BIO X bioprinter (CELLINK). Two composite blends (Formulation A and Formulation B) were tested under controlled laboratory conditions (23 ± 2 °C, 1 atm). The extrusion process was carried out at 37 °C (physiological temperature), with the print bed temperature also set to 37 °C.

#### 2.6.1 Bridge Test

To assess the self-supporting capability of the hydrogels, a “bridge test” was performed. This involved extruding filaments over support structures with increasing spans between pillars (from 1 mm to 7 mm, in regular steps). Printing was performed with a BIO X bioprinter (CELLINK), using a 22G conical nozzle, at a speed of 4 mm·s^–1^ and constant pneumatic pressure of 44 kPa.

After printing, filaments were visually inspected and qualitatively classified as “complete collapse” or “no collapse”. For a more objective assessment, the actual area occupied by the filament suspended between two pillars was measured and compared with the theoretical span area. If the filament occupied more than 50% of the theoretical area, the hydrogel was considered to have failed the test for that span. A 2 min waiting period was observed before evaluation to prevent misjudgments due to the material’s initial fluidity.

#### 2.6.2 Patch Test

The geometric fidelity of the formulations was evaluated through a mesh printing assay (“patch test”), designed to simulate patch-like structures. A CAD pattern with uniform square geometry was used, over which the bioinks were printed under three controlled combinations of print speed (*v*) and pneumatic pressure (*P*)­

*v* = 4 mm·s^–1^, *P* = 44 kPa
*v* = 8 mm·s^–1^, *P* = 57 kPa
*v* = 12 mm·s^–1^, *P* = 66 kPa

After deposition, the structures were photographed after 2 min. The resulting images were analyzed using AutoCAD 2023 (Autodesk Inc.), determining the internal area of each printed cell and comparing it to the theoretical area of the digital model. Print fidelity was quantified as the percentage match between the theoretical (CAD model) and actual (printed structure) areas, averaging the values of all the cells in each printed patch.

## 3 Results and Discussion

This section presents and analyzes the results obtained from the tests performed to characterize the rheological properties, cytotoxicity, and printability of the developed polysaccharide-based bioinks. The collected data allow for the evaluation of the behavior of the polysaccharide solutions and their suitability for 3D bioprinting, providing a solid foundation for optimizing the formulations. Below, the results of the rheological tests are detailed, which represent the first stage of characterization and lay the groundwork for understanding the viscoelastic properties of the individual components and their performance in blended formulations.

### 3.1 Rheological Test of Polysaccharide Solutions

Rheological characterization of the polysaccharide solutions (KC, TG, and KG) was carried out to evaluate their behavior under mechanical stress and their response to flow, an essential step prior to analyzing the combined formulations. This initial stage enables the identification of the intrinsic viscoelastic properties of each component individually, providing key information for understanding their contribution in mixtures and optimizing the final formulation of the bioinks.

The rheological tests included the determination of the storage modulus (*G*′) and loss modulus (*G*″), flow curves under controlled shear, and thermal sweeps. This set of analyses enabled the evaluation of each polysaccharide’s structural stability, pseudoplastic behavior, and thermal sensitivity of the solutions to ensure controlled extrusion and structural fidelity during three-dimensional bioprinting.

#### 3.1.1 Kappa-Carrageenan

The rheological analyses of the stock solutions of Kappa-Carrageenan (KC) at different concentrations are shown in Figure 2.

**Figure 2:**
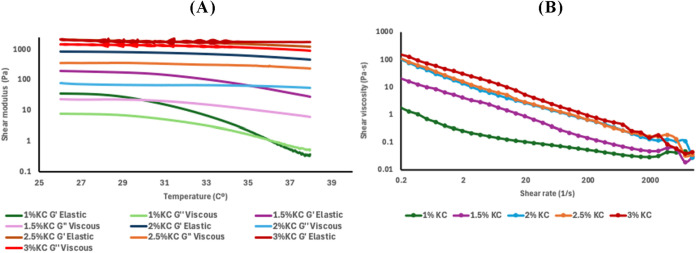
Rheological analysis of stock solutions of κ-carrageenan (KC) at different concentrations. (A) Evolution of viscoelastic moduli G′ (elastic) and G″ (viscous) as a function of temperature (26–38 °C). (B) Apparent viscosity behavior as a function of shear rate at 37 °C (range 0.2–1000 s^–1^).

The rheological tests performed on κ-carrageenan (KC) solutions revealed a dominant viscoelastic behavior, with a clear prevalence of the storage modulus (*G*′) over the loss modulus (*G*″) throughout the evaluated temperature range (Figure 2A). This dominance of *G*′ indicates the formation of a solid-like, three-dimensional network, even under physiological conditions,^44,45^ consistent with the findings of Campo et al., who reported that the helical structure of κ-carrageenan chains promotes the formation of thermostable gels with tunable mechanical properties.^16^

An increase in KC concentration led to a progressive rise in both viscoelastic moduli, with *G*′ values exceeding 1000 Pa at concentrations ≥ 2% (w/v). This behavior aligns with the results of Van de Velde et al., who showed that higher carrageenan concentrations induce a greater density of hydrogen bonds and the formation of helical junction zones, thereby strengthening the hydrogel network.^46^ Notably, the 3% formulation exhibited the highest structural stability, with no crossover between *G*′ and *G*″ in the evaluated temperature range, indicating a robust network that maintains its integrity without a gel-to-sol transition. This result is comparable to what was reported by Li et al., who emphasized that concentrations above 2% are suitable for bioprinting applications, as they provide sufficient stiffness to maintain shape fidelity after extrusion.^47^

In contrast, the 1 and 1.5% KC formulations showed a significant decrease in *G*′ starting at 32 °C, with the 1% solution displaying a gel-to-sol transition around 36 °C, marked by the crossover of *G*′ and *G*″. This phenomenon reflects reduced thermal stability and a loss of structural cohesion under physiological conditions, potentially compromising their use as structural bioinks in bioprinting processes, as also noted by Schütz et al. for polysaccharide-based bioinks with insufficient gel network concentration.^48^

In the flow tests (Figure 2B), all formulations exhibited pseudoplastic behavior typical of polysaccharides, characterized by a progressive decrease in apparent viscosity with increasing shear rate.^49,50^ This behavior is crucial for extrusion-based bioprinting, as it facilitates controlled hydrogel deposition and enables rapid structural recovery after passing through the nozzle, thereby preserving the fidelity of the printed geometry.^51–54^ This phenomenon, also known as *shear-thinning*, is common in hydrogels based on polysaccharides such as Alginate, Gellan Gum, Agarose, and Cellulose derivatives. It is particularly advantageous because it reduces the pressure needed for extrusion and minimizes cell damage during the process.^51–53,55^

Moreover, the ability of polysaccharide hydrogels to recover their structure after extrusion contributes to the stability and precision of printed constructs, which is essential for applications in tissue engineering and tumor modeling.^52,53,55,56^ The rheological properties, such as viscosity and elasticity, can be tuned by combining different polysaccharides, incorporating rheology modifiers like nanocellulose, or adjusting cell density and the concentration of ions or nanoparticles.^51,54,55,57^

The κ-carrageenan (KC) formulations demonstrated a pseudoplastic behavior that meets the requirements for efficient 3D bioprinting, enabling the fabrication of biomimetic structures with high cell viability and geometric Fidelity.^51–56^ None of the formulations exhibited excessively high initial viscosity (>10^4^ mPa·s at low shear rates), ensuring stable flow during the printing process. This balance between moderate initial viscosity and a pseudoplastic profile is consistent with previous studies highlighting the ability of KC-based bioinks to combine printability and postprinting stability.^58–62^

Overall, the results confirm that KC formulations at concentrations equal to or greater than 2% possess optimal rheological properties for 3D bioprinting, including high thermal stability, sufficient structural rigidity to maintain shape after deposition, and ideal pseudoplastic behavior for extrusion.

#### 3.1.2 Tragacanth Gum

The rheological analyses of tragacanth gum (TG) solutions at different concentrations are presented in Figure 3.

**Figure 3:**
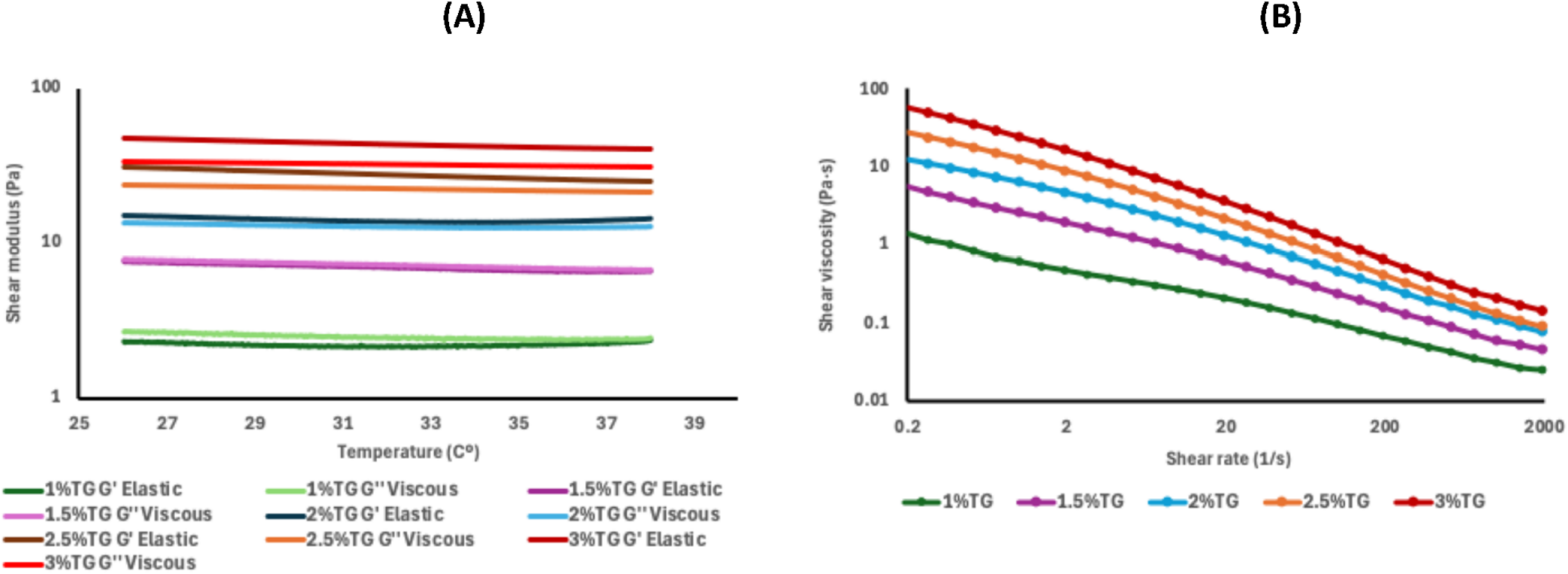
Rheological analysis of stock solutions of tragacanth gum (TG) at different concentrations. (A) Evolution of the viscoelastic moduli G′ (elastic) and G″ (viscous) as a function of temperature (26–38 °C). (B) Apparent viscosity behavior as a function of shear rate at 37 °C (range: 0.2–2000 s^–1^).

As shown in Figure 3A, the TG solutions exhibited a viscoelastic profile characterized by stable storage (*G*′) and loss (*G*″) moduli within the evaluated temperature range (26–38 °C). At all concentrations tested, *G*′ consistently remained higher than *G*″, indicating a predominantly solid-like elastic gel behavior. However, the absolute values of *G*′ were notably lower than those observed for κ-carrageenan, suggesting a less dense structural network with lower resistance to deformation.

Increasing the TG concentration led to a gradual rise in both moduli, which became more pronounced at concentrations ≥2% (w/v). Despite this increase, the values did not reach those typical of strongly cross-linked materials. Importantly, none of the formulations showed a crossover between *G*′ and *G*″ within the 26–38 °C range, indicating suitable thermal stability for applications at physiological temperature. These findings align with those reported by Balaghi et al.,^22^ who documented that aqueous tragacanth gum solutions display *G*′ values between 1 and 100 Pa, depending on botanical source and concentration, and maintain stable behavior without sharp thermal transitions under similar conditions.

Despite its lower structural stiffness, this rheological response may be advantageous in formulations requiring flexibility or a low elastic modulus, such as controlled release matrices or soft gels for cellular contact.^22^ Additionally, its thermal stability and ability to maintain *G*′ > *G*″ even at low concentrations enhance its potential as a stabilizing agent in multicomponent systems with other biopolymers.^63^

Regarding flow behavior, the tests shown in Figure 3B demonstrated that all TG concentrations exhibited a markedly pseudoplastic profile, characterized by a continuous decrease in apparent viscosity with increasing shear rate. This behavior, common in polysaccharides such as xanthan gum or gum arabic,^63^ is highly desirable for bioprinting processes, as it facilitates pressure-driven extrusion without compromising postprinting structural integrity. Formulations with TG concentrations ≥2% (w/v) displayed sufficiently high initial viscosity to maintain the printed structures’ shape without collapse, whereas the 1% (w/v) formulation was excessively fluid for this purpose.

In summary, the results indicate that tragacanth gum can be integrated as a secondary structural component in bioinks, contributing thermal stability and favorable pseudoplastic behavior, particularly at concentrations equal to or above 2% (w/v). However, its lower stiffness compared to κ-carrageenan limits its application as a standalone component in self-supporting formulations, making it more suitable for multicomponent systems where it functions as a rheological modifier.

#### 3.1.3 Konjac Glucomannan

The rheological analyses shown in Figure 4 for KG solutions display a viscoelastic profile characterized by high structural stiffness, even at low concentrations (Figure 4A). Under all tested conditions, the storage modulus (*G*′) consistently exceeded the loss modulus (*G*″), reflecting predominantly elastic behavior and notable stability against thermal variations in the 26–38 °C range. This pattern is consistent with the findings reported by Huang et al.,^64^ who noted that the addition of KG significantly enhances the elasticity of various formulations.

**Figure 4:**
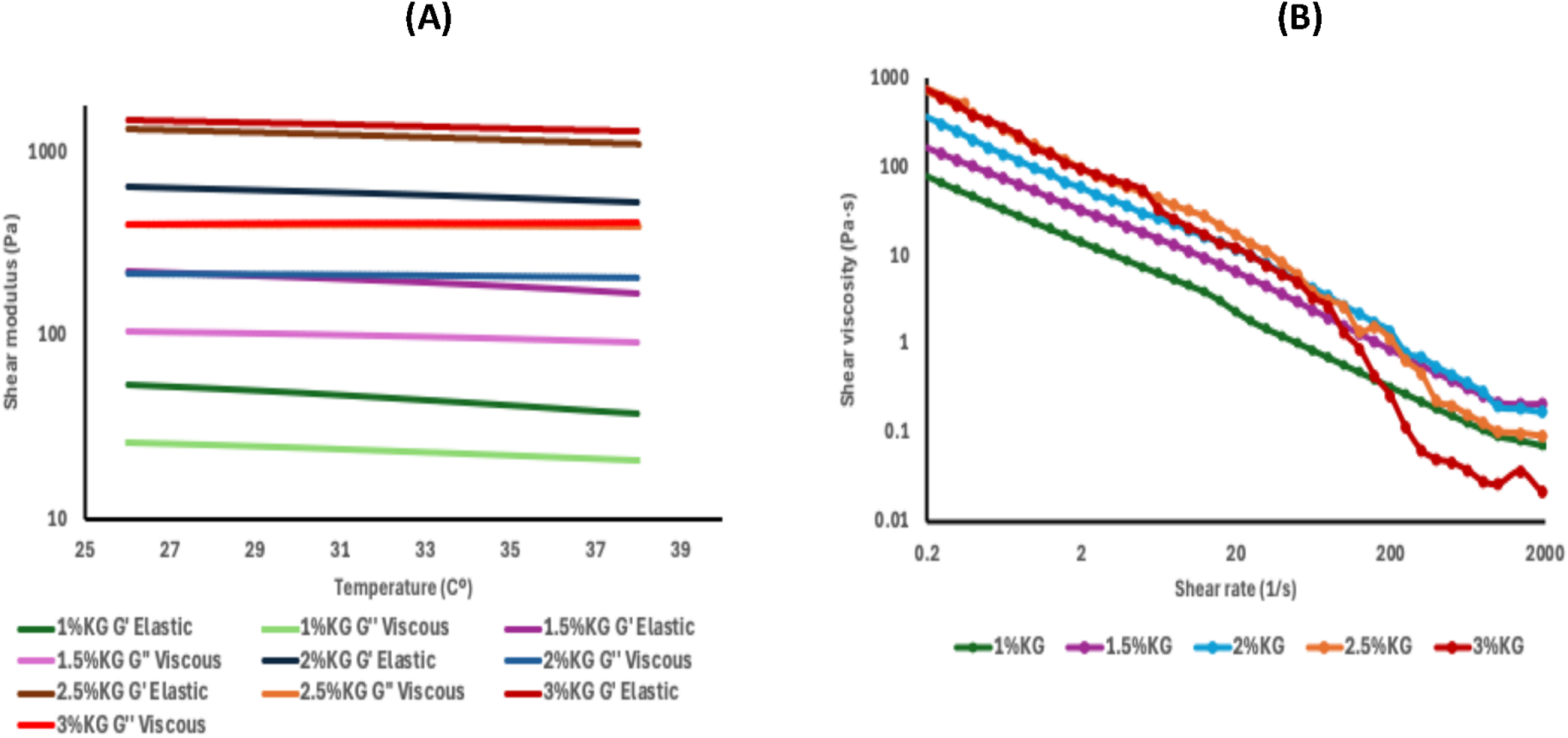
Rheological analysis of stock solutions of konjac glucomannan (KG) at different concentrations. (A) Evolution of the viscoelastic moduli *G*′ (elastic) and *G*″ (viscous) as a function of temperature (26–38 °C). (B) Apparent viscosity behavior as a function of shear rate at 37 °C (range 0.2–2000 s^–1^).

An increase in KG concentration led to a progressive rise in both moduli, particularly *G*′, which exceeded 1000 Pa in the 3% (w/v) formulation. This value is comparable to that observed for κ-carrageenan at the same concentration, although KG displayed lower thermal sensitivity; the *G*′ and *G*″ curves for all formulations remained nearly parallel and without crossover throughout the tested temperature range (26–38 °C). This indicates a robust and thermally stable viscoelastic network, a well-known characteristic of konjac glucomannan.^65–67^ Such stability is attributed to the formation of extensive junction zones and the ability of KG to induce a compact and regulated gel network, reinforced by hydrogen bonding and volume exclusion effects.^65,66,68^ Additionally, the incorporation of KG into mixed systems, such as with κ-carrageenan, improves elasticity, cohesiveness, water retention capacity, and thermal stability of the gels.^68,69^

In flow tests (Figure 4B), all KG formulations exhibited a clear pseudoplastic behavior, with a decrease in apparent viscosity as shear rate increased. This shear-thinning behavior is an intrinsic property of konjac glucomannan and has been widely reported in the literature.^70–72^ The initial viscosity of KG solutions increases proportionally with concentration, easily exceeding 1000 Pa·s in 3% (w/v) hydrogels, while intermediate concentrations (1.5–2% w/v) offer a good balance between structural stability and ease of extrusion, particularly relevant for applications such as bioprinting and 3D food printing.^70,73^ On a molecular level, shear thinning is explained by the alignment and subsequent collapse of macromolecular chains under high shear rates, which reduces flow resistance.^71^ Moreover, viscosity and rheological behavior can be modulated by factors such as temperature, salt presence, and interactions with other biopolymers, allowing the tuning of hydrogel properties for specific applications.^71,73,74^

In summary, konjac glucomannan exhibits a robust rheological profile, characterized by high elasticity and considerable thermal resistance. However, its high viscosity at greater concentrations underscores the need for careful optimization of processing parameters to ensure a continuous and stable flow in relevant applications.^70,71,73^

### 3.2 Cytotoxicity Assay

Cytotoxicity assessment is essential for determining the biocompatibility of materials intended for biomedical applications, as it helps identify potential adverse effects on cell viability. The MTS assay is a widely accepted method that quantifies metabolically active cells, providing a direct measure of cell viability and, consequently, biocompatibility.^75–77^ According to ISO 10993–5, a biomaterial is considered biocompatible if cell viability exceeds 70%. This threshold is an international standard in the evaluation of new materials.^75,78^ Previous studies have demonstrated that materials such as calcium silicate cements, hydrogels, and nanomaterials exhibit low cytotoxicity and high biocompatibility when meeting this criterion, making them suitable for clinical applications.^75–77^

However, factors such as chemical composition, particle size, and the presence of impurities or synthesis residues can influence cytotoxicity. For instance, in nanomaterials like graphene and MXene, toxic residues may induce cell death, complicating data interpretation.^79,80^ Additionally, the methodology employed and the cell type used in the assays can impact the results, underscoring the need for standardized protocols to ensure consistent and reproducible evaluations.^78,81^ Therefore, rigorous cytotoxicity testing using methods like the MTS assay, in compliance with recognized standards, is critical for ensuring the safety and efficacy of biomaterials before clinical application.^75–78^

#### 3.2.1 Cytotoxicity Analysis of KC Hydrogel

The cytotoxicity analysis of KC hydrogels (Figure 5) shows that higher concentrations of this polymer are generally associated with increased cell viability, suggesting good biocompatibility under proper formulation conditions. These results are consistent with several studies reporting that, within the first 24 h of exposure, a significant reduction in cell viability, approaching 50%, can occur, particularly at low concentrations. In contrast, concentrations equal to or greater than 2% (w/v) tend to maintain or even enhance viability. After 48 h of culture, most samples at concentrations ≥2% (w/v) exhibited cell viability above 85%. These findings support the notion that KC hydrogels, when used at appropriate concentrations, are safe and cell-compatible materials, making them suitable for biomedical and tissue engineering applications. However, careful evaluation of concentration and exposure time is crucial to minimize initial cytotoxic effects and promote long-term cellular recovery.^82^

**Figure 5:**
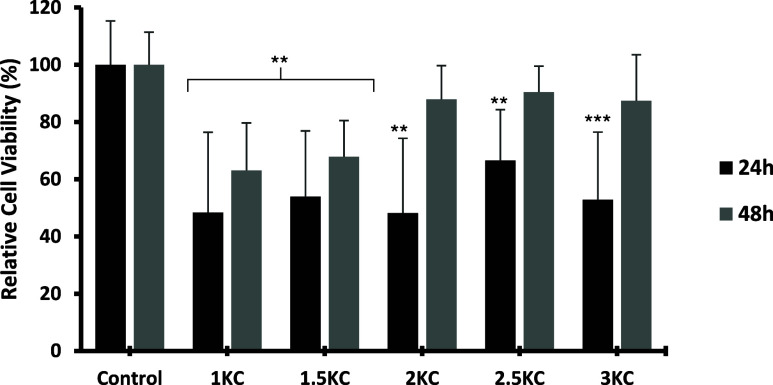
Relative Cell Viability percentages at 24 and 48 h of culture for different concentrations of KC, with labels corresponding to those described in Table 1. ANOVA test results indicated high statistical significance both 24 and 48 h (***), and post hoc comparisons between the control and each treatment group were performed using Student’s *t* test. Statistical significance was considered at *p* < 0.05 (*), *p* < 0.01 (**), and *p* < 0.001 (***).

#### 3.2.2 Cytotoxicity Analysis of TG Hydrogel

The cytotoxicity analysis of TG hydrogel (Figure 6) revealed a significant decrease in cell viability during the first 24 h of culture, particularly at 1 and 1.5% (w/v) concentrations, which showed viabilities of 42.76 ± 23.4 and 57.53 ± 28.7%, respectively. Higher concentrations (2, 2.5, and 3%) reached values close to 60%, all falling below the 70% noncytotoxicity threshold recommended for biomedical materials.^83^ However, at 48 h, a generalized recovery in cell viability was observed, with average increases of approximately 30%. This recovery was especially notable at the 4% concentration, which experienced an increase of nearly 40 percentage points.

**Figure 6:**
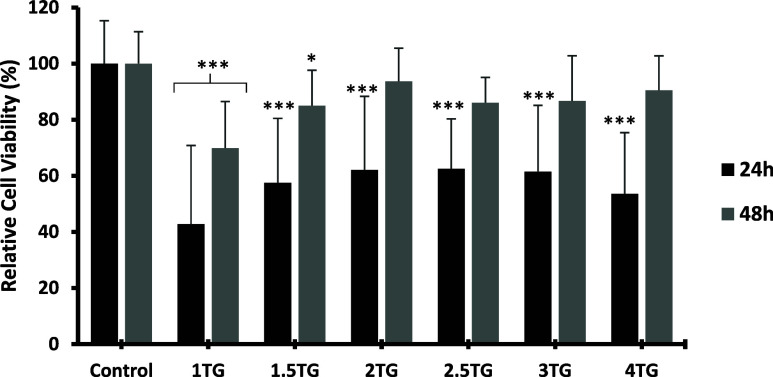
Relative cell viability percentages after 24 and 48 h of culture for different TG concentrations, whose label names are referenced in Table 1. ANOVA test results indicated high statistical significance both 24 and 48 h (***), and post hoc comparisons between the control and each treatment group were performed using Student’s *t* test. Statistical significance was considered at *p* < 0.05 (*), *p* < 0.01 (**), and *p* < 0.001 (***).

After this period, all concentrations, except the 1% (w/v), surpassed 85% viability. The 2 and 4% formulations exhibited the highest values (93.70 ± 16.8 and 90.48 ± 7.1%, respectively), indicating good biocompatibility following the initial adaptation passage.^84,85^ This pattern of early reduction followed by recovery can be attributed to cellular responses to the novel hydrophilic-viscoelastic microenvironment of the hydrogel, which activates stress and adaptation pathways before metabolic activity returns to baseline levels.^85,86^

Previous studies have demonstrated that the physicochemical composition of the hydrogel, the type and concentration of cross-linking agents, and the presence of synthesis residues significantly influence cell viability and adaptive responses.^82,86,87^ Therefore, the observed recovery suggests that, after an initial adjustment period, TG hydrogels can be considered biocompatible materials for biomedical applications—provided that synthesis conditions and concentration are optimized to minimize early cytotoxic effects.^84,85^

#### 3.2.3 Cytotoxicity Analysis of KG Hydrogel

Regarding cell cultures with hydrogels formulated with different concentrations of KG (Figure 7a) marked decrease in cell viability was observed at the 1% (w/v) concentration, with values of 31.3 ± 8.6% at 24 h and 28.2 ± 5.25% at 48 h, indicating significant cytotoxicity. In contrast, the 2.5 and 3% concentrations exhibited high viability levels at 24 h (80.19 ± 23.4 and 81.8 ± 13.0%, respectively), followed by a considerable reduction of more than 20 percentage points at 48 h (55.3 ± 9.8 and 60.7 ± 10.3%, respectively), falling below the biocompatibility threshold.

**Figure 7:**
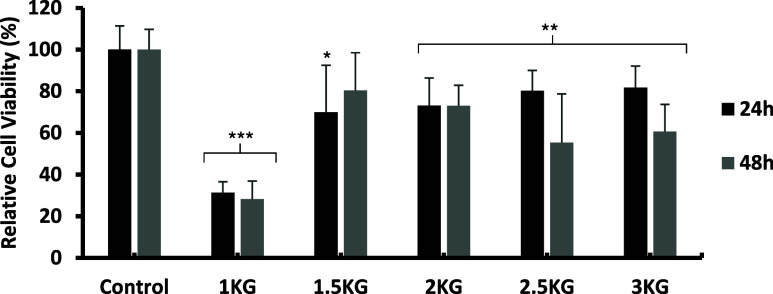
Relative cell viability after 24 and 48 h of culture for different KG concentrations, with label names referenced in Table 1. ANOVA test results indicated high statistical significance both 24 and 48 h (***), and post hoc comparisons between the control and each treatment group were performed using Student’s *t* test. Statistical significance was considered at *p* < 0.05 (*), *p* < 0.01 (**), and *p* < 0.001 (***).

In this context, only the 1.5 and 2% concentrations were found to be suitable in terms of biocompatibility. The former showed a positive trend, with an increase in cell viability from 69.9 ± 18.0% at 24 h to 80.45 ± 22.6% at 48 h. Meanwhile, the 2% concentration maintained a stable cell viability of around 73% throughout the culture period, consistently meeting the criteria established to consider the material biocompatible.

The decrease in cell viability observed with KG concentrations above 1.5% could be mainly attributed to the increased viscosity of the medium, which limits the diffusion of nutrients and oxygen. Several studies have shown that increasing hydrogel concentration, and therefore viscosity, reduces the diffusion coefficient of oxygen and nutrients,^88^ decreases cell proliferation, and restricts water availability for cellular metabolism.^23,89^

Regarding the low viability observed at the 1% KG concentration, the literature suggests two nonmutually exclusive mechanisms. On one hand, a low KG concentration may imply a low degree of cross-linking, leaving functional groups exposed with high hydrogen bonding capacity, which could promote nonspecific adsorption of essential medium components, such as proteins and other ligands, thus reducing their availability to cells.^23^ On the other hand, during thermal sterilization (e.g., autoclaving), potentially cytotoxic soluble compounds may be released. Among these, the release of acetyl groups has been reported; their presence in the medium can induce cellular stress through nonspecific acetylation of proteins,^44,90,91^ thereby disrupting critical functions for cellular homeostasis. In the specific case of KG, it has been reported that changes in temperature and exposure to an alkaline environment can lead to deacetylation,^92^ however, it remains unclear whether alkalinization of the medium occurred during the experiment.

### 3.3 Selection and Characterization of the Final Formulations

The selection and characterization process of the final formulations was first carried out based on the parameters obtained during the individual characterization of the base hydrogels, evaluating their rheological and biological properties to determine the optimal concentration ranges of each polysaccharide. Subsequently, blends of these hydrogels were designed, carefully adjusting the concentrations to combine the advantages of each component and overcome the limitations of the individual hydrogels.

The objective of this process was to develop formulations that improve the mechanical performance, stability, and biocompatibility of each individual hydrogel, achieving a matrix with suitable properties for advanced applications such as 3D bioprinting. These blends allow for optimization of viscoelasticity and cell viability, ensuring structural integrity during the printing process and subsequent functionality in contact with living tissues.

#### 3.3.1 Selection of the Final Formulations

The selection process for the final polysaccharide-based hydrogel formulations was based on the search for an optimal balance between mechanical properties, stability, and biocompatibility; key aspects for advanced biomedical applications such as tissue engineering and bioprinting. The choice of a minimum concentration of 2% κ-carrageenan (KC) is supported by studies showing that this level significantly improves the strength and stability of hydrogels without affecting biocompatibility, which is crucial to avoid adverse cellular responses and ensure functionality in contact with living tissues.^93,94^ For tragacanth gum (TG), a concentration of 4% was selected, as previous research has shown that this range maximizes cell viability and enhances mechanical properties, critical factors for withstanding the physiological environment and promoting tissue regeneration.^94^ As for konjac glucomannan (KG), concentrations between 1.5% and 2% were evaluated, consistent with the literature indicating that these values yield gels with optimal texture and stability, while remaining cell-compatible.^93,94^

The strategy of combining and adjusting these components aligns with recent approaches emphasizing the importance of formulation to optimize both mechanical strength and biocompatibility in natural hydrogels, thereby overcoming the traditional limitations of pure polymers.^94,95^ In summary, the selection of formulations A (2% KC, 4% TG, 1.5% KG) and B (2% KC, 4% TG, 2% KG) (Table 2) responds to the need to compare the impact of KG on overall performance and identify which composition offers the best integrated properties in terms of viscoelasticity, cell stability, and suitability for bioprinting.

**Table 2:** Composition of the Selected Formulations

selected formula A		selected formula B	
tag	“formula A”	tag	“formula B”
composition	2% KC, 4% TG and 1.5% KG	composition	2% KC, 4% TG and 2% KG

#### 3.3.2 Rheological Tests

Rheological tests are essential for evaluating the suitability of formulations A and B intended for bioprinting, as they allow for the analysis of viscoelastic behavior and flow response under conditions relevant to the 3D printing of hydrogels. The rheological analysis of the formulations is presented in Figure 8.

**Figure 8:**
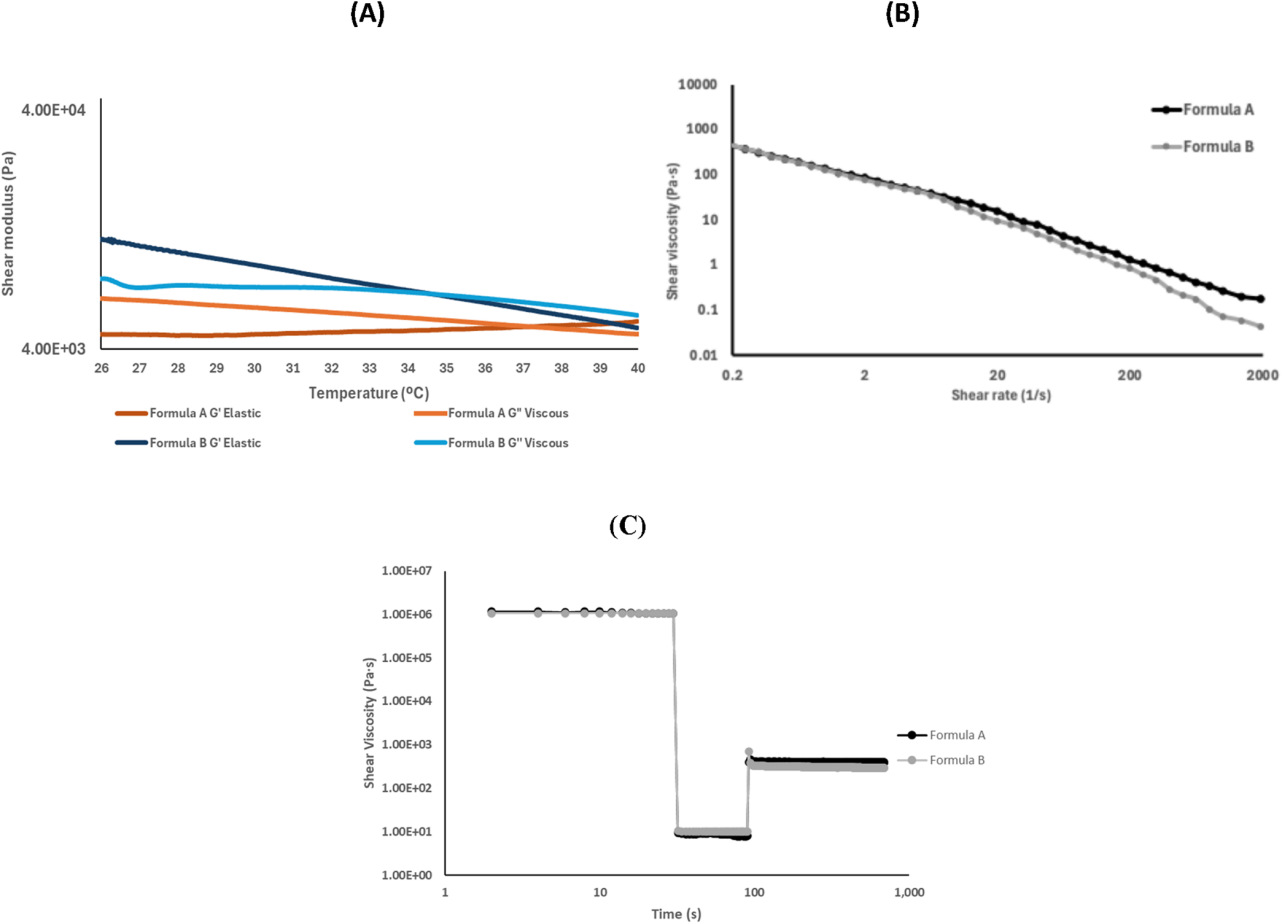
Rheological analysis of the formulation. (A) Viscoelastic moduli *G*′ (elastic) and *G*″ (viscous) as a function of temperature (26–40 °C); (B) apparent viscosity versus shear rate at 37 °C (0.2–2000 s^–1^); (C) three-interval thixotropy test (3ITT) for Formulations A and B performed at 37 °C. The test consisted of three consecutive steps: an initial low-shear interval at 0.1 s^–1^ for 5 s, a high-shear interval at 150 s^–1^ for 60 s simulating extrusion, and a final low-shear interval identical to the first step.

To assess the structural rebuilding capacity of the formulations after shear-induced disruption, a three-interval thixotropy test was performed at 37 °C (Figure 8). Both formulations exhibited a pronounced decrease in viscosity from approximately 1.0 × 10^6^ Pa·s at low shear to around 9 Pa·s during the high-shear interval, which mimics the extrusion process. Upon returning to low-shear conditions, the viscosity rapidly increased and stabilized at approximately 400 Pa·s for Formulation A and 300 Pa·s for Formulation B, corresponding to a recovery of nearly 40% of the initial value. This rapid and stable recovery at physiological temperature indicates effective reformation of the hydrogel network after extrusion. Such thixotropic behavior is essential in extrusion-based bioprinting since it allows the material to flow easily through the nozzle and subsequently regain sufficient structural integrity to maintain the printed shape.

Both formulations exhibit stable solid-elastic behavior and pseudoplastic properties, which are desirable characteristics because they facilitate extrusion and help maintain shape after deposition, essential for the geometric fidelity of printed structures.^96–99^ However, relevant differences were observed in structural stiffness and flow response: Formulation B showed greater stiffness, while Formulation A exhibited higher initial viscosity.

In the thermal tests (Figure 8A), Formulation B maintained a storage modulus (*G*′) higher than the loss modulus (*G*″) from 26 °C up to approximately 34.5 °C, suggesting a more robust polymer network, which is associated with better structural stability during and after printing.^96,100,101^ However, the inversion of *G*′ and *G*″ near physiological temperature indicates a potential weakening of the network, a phenomenon reported in other hydrogels that may compromise print fidelity under physiological conditions.^96,99^

On the other hand, Formulation A, with a lower concentration of gelling agent, showed a more viscous profile and a less defined network, which aligns with studies highlighting the importance of polymer concentration in forming solid three-dimensional networks.^99,101,102^ Altogether, the data confirm that KG concentration is a key factor in the mechanical balance of these multicomponent formulations.

In the flow tests (Figure 8B), both formulations showed pseudoplastic behavior, with a decrease in viscosity as shear rate increased. This is crucial for controlled extrusion and cell viability, as it reduces stress on cells during the process.^96,98,101,102^

Formulation A exhibited slightly higher initial viscosity, which could help control flow during extrusion at 37 °C. However, Formulation B maintained a more stable and balanced profile, combining appropriate viscosity with better mechanical properties at temperatures close to physiological, making it more promising for bioprinting processes that require structural stability after deposition. Taken together, the results suggest that both formulations are technically printable, but Formulation B offers a more robust combination of structural stiffness, pseudoplastic behavior, and thermal stability under physiological conditions.

Finally, the enhanced viscoelastic behavior of the multicomponent formulations can be rationalized by the molecular interactions occurring among KC, TG and KG. KC contributes a highly sulfated backbone that forms double-helical aggregates stabilized by electrostatic screening in the presence of K^+^ ions from PBS.^16,17^ TG introduces additional anionic and carboxylic groups capable of partially screening the negative charge density of KC chains, reducing repulsive forces and facilitating closer chain packing. Simultaneously, TG’s abundant hydroxyl functionalities promote intermolecular hydrogen bonding, which acts as reversible physical cross-linking sites that reinforce the three-dimensional network.^32,33^ KG further enhances this cooperative network through multiple hydrogen bonds and van der Waals interactions, while its flexible β-1,3 linkages and partial acetylation favor chain mobility and entanglement, enabling efficient stress dissipation under deformation.^36,39,40^ The synergistic contribution of ionic screening (KC–TG) and hydrogen bonding (TG–KG–KC) therefore explains the higher storage modulus (*G*′) and improved structural stability observed in the blended systems, particularly in Formulation B, where increased KG concentration provides additional cross-linking flexibility without disrupting the integrity of the KC-dominated gel network.

The rheological behavior of the KC–TG–KG system was compared with that commonly reported in other bioinks. Under physiological temperature conditions (37 °C) rheological profile is consistent with that observed in other formulations, where a strong elastic component contributes to shape retention after extrusion and supports structural stability during the printing process.^103^

In addition, the formulations displayed a pronounced shear-thinning behavior, characterized by a marked decrease in apparent viscosity as the shear rate increased. Such pseudoplastic flow is a well-established feature of extrusion-printable bioinks and has been widely documented for alginate and GelMA systems (Cooke et al.; Elango et al.). This behavior facilitates controlled material extrusion through fine nozzles, reduces printing pressures, and minimizes shear-induced stress on cells, which are critical parameters for maintaining print fidelity and cell viability. Altogether, these rheological features position the KC–TG–KG system within the expected performance envelope of standard bioinks used in extrusion-based bioprinting.^104–106^

#### 3.3.3 Cytotoxicity Assay

The cytotoxicity assay conducted for the selected formulations A and B (Figure 9) showed that both maintained acceptable cell viability in accordance with ISO 10993–5. After 24 h of culture, Formulation A presented a cell viability of 86.51 ± 13.92%, while Formulation B recorded 72.68 ± 9.18%. At 48 h, cell viability remained relatively stable: 82.14 ± 3.13% for Formulation A and 72.83 ± 4.47% for Formulation B. In both cases, values remained above the minimum threshold of 70%, confirming their biocompatibility and cellular stability during the evaluation period.

**Figure 9:**
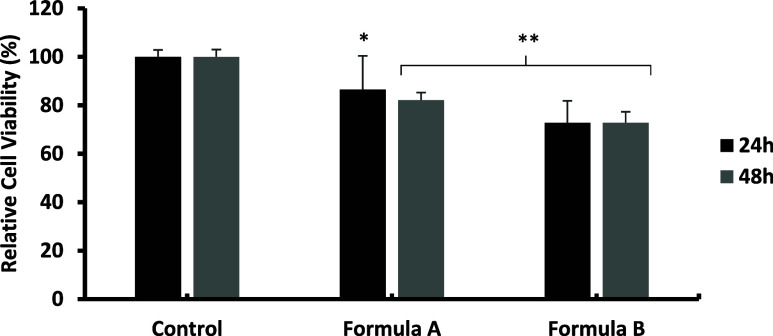
Relative cell viability compared to the control after 24 and 48 h of culture for formulations A and B. ANOVA test results indicated high statistical significance both 24 and 48 h (***), and post hoc comparisons between the control and each treatment group were performed using Student’s *t* test. Statistical significance was considered at *p* < 0.05 (*), *p* < 0.01 (**), and *p* < 0.001 (***).

These results contrast with those obtained for κ-carrageenan (KC) and tragacanth gum (TG) solutions, which showed increases in cell viability of between 30 and 40% over the same period. In contrast, konjac glucomannan (KG) exhibited a pattern similar to that of formulations A and B, with moderate cell viability. This difference could be due to modifications in the mechanical and structural properties of the microenvironment created by the formulated hydrogels, which affect key processes such as cell adhesion, proliferation, and survival.^107,108^

To optimize these results, it is suggested to incorporate adhesion peptides, growth factors, or other bioactive molecules that enhance cell integration and hydrogel functionality. For example, peptide sequences such as RGD (Arg-Gly-Asp) have been shown to significantly improve cell adhesion by mimicking extracellular matrix binding sites.^109^ Likewise, growth factors like VEGF (Vascular Endothelial Growth Factor) can stimulate angiogenesis and facilitate nutrient diffusion through the scaffold.^110^

Overall, the results suggest that both formulations are technically printable, but Formulation B offers a more robust combination of structural stiffness, pseudoplastic behavior, and thermal stability under physiological conditions.

#### 3.3.4 Printability Test

The printability of formulations A and B was evaluated using two complementary tests: the bridging test and the patch test. The objective was to analyze each hydrogel’s ability to maintain self-supporting structures and accurately reproduce complex geometric patterns with high dimensional fidelity.

##### 3.3.4.1 Bridging Test

The bridging test is a fundamental method for assessing a hydrogel’s self-supporting capability during extrusion and layer-by-layer deposition. This test allows for a direct analysis of the immediate mechanical strength of the formed filaments and their ability to support their own weight between pillars without collapsing, which is critical to ensuring the dimensional stability of more complex printed structures. The following section discusses the results obtained for formulations A and B, considering their relationship to rheological properties and their suitability for bioprinting applications.

In the bridging test (Figure 10), the ability of each hydrogel to support extruded filaments over progressively spaced pillars (1–7 mm) was evaluated at a controlled temperature between 36–37 °C. Formulation B was able to maintain stable filaments up to a 6 mm gap between pillars without collapsing, whereas Formulation A showed breakage or deformation starting at 4–5 mm. These results are consistent with the previously observed rheological properties, indicating that Formulation B possesses a stiffer and more stable network, with sufficient flow resistance to withstand gravitational collapse after deposition.

**Figure 10:**
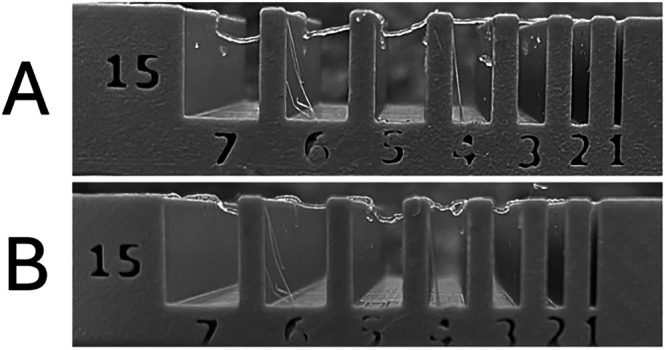
Bridging test of formulations A and B. The ability of filaments to span between pillars up to 7 mm apart is shown. Formulation B supports intact filaments up to 6 mm without collapse, while Formulation A exhibits structural failures starting at 5 mm.

##### 3.3.4.2 Patch Test

The patch test was designed to assess the geometric fidelity of each formulation under real 3D printing conditions. This test determines the hydrogel’s ability to reproduce complex lattice patterns with high dimensional accuracy and structural consistency, key factors for bioprinting applications, where the accuracy of the printed shape directly impacts the functionality of the final construct. The following section analyzes the results obtained for formulations A and B, relating them to their extrusion properties and stability during deposition.

The patch test (Figure 11 and Table 3) allowed for the quantification of geometric fidelity under real 3D printing conditions. Lattice structures with a known geometry were printed under three combinations of printing speed and pneumatic pressure: (*X*.1): 4 mm/s and 44 kPa;, (*X*.2): 8 mm/s and 57 kPa;, (*X*.3): 12 mm/s and 66 kPa, where *X* corresponds to the formulation (A or B).

**Figure 11:**
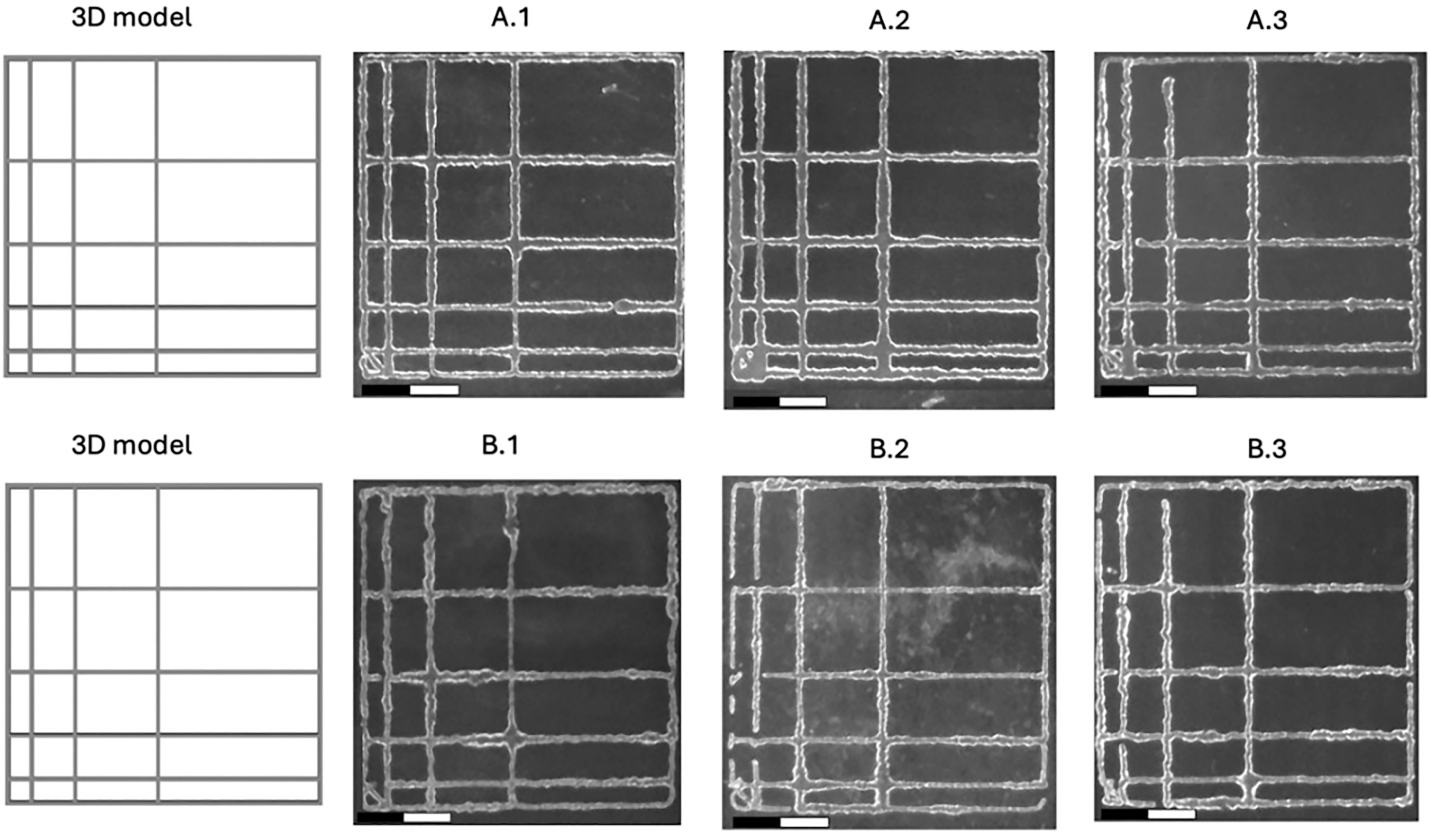
Comparison between the CAD model and the lattice structures printed with formulations A and B under three printing conditions: A.1/B.1:4 mm/s – 44 kPa; A.2/B.2:8 mm/s – 57 kPa; A.3/B.3:12 mm/s – 66 kPa. The scale bar represents 10 mm. Greater definition and structural fidelity were observed in Formulation B.

**Table 3:** Relationship between the Fidelity of the Printed Lattice and the CAD Model^a^

A.1	(%)	average	81.73	A.2	(%)	average	73.48	A.3	(%)	average	79.09
72.71	84.24	87.14	90.85	65.63	82.33	89.29	95.28	76.35	87.05	92.89	95.17
70.45	81.71	84.84	87.05	57.42	81.17	82.56	92.70	70.10	86.36	87.55	91.85
73.00	80.92	86.57	82.20	61.00	78.12	81.21	85.72	65.00	81.62	82.77	87.42
70.66	79.50	89.19	82.31	52.74	70.06	78.88	78.32	63.93	77.88	78.96	83.05
Null	72.07	72.83	104.55	Null	52.87	51.79	59.05	Null	57.01	64.27	73.45

B.1	(%)	average	84.66	B.2	(%)	average	84.82	B.3	(%)	average	84.32
83.10	86.04	91.21	93.17	91.93	88.54	93.39	96.36	88.34	90.19	93.17	96.93
83.37	87.52	88.71	95.03	Null	87.40	90.77	94.68	74.86	84.20	90.41	93.30
77.15	80.41	80.67	91.39	Null	87.99	87.49	89.00	71.77	88.96	87.01	90.91
Null	87.55	86.58	87.59	83.79	82.74	78.24	83.18	76.26	86.28	81.66	89.09
Null	71.26	74.65	78.49	Null	70.23	57.80	78.37	Null	79.77	63.98	75.04

a Each rectangle represents a specific area of the patch analyzed. Data are expressed as a percentage match relative to the original design. Cells with a “Null” value indicate areas with severe defects or measurement failures that were excluded from the analysis. The underlined cells correspond to areas with positioning defects; however, their closure was simulated to estimate the area-to-area match with the CAD model, allowing their inclusion in the fidelity calculation.

The analyses showed (Table 3 and Figure 12) that both formulations were able to accurately reproduce the target geometry, although with clear differences in consistency and structural definition.

**Figure 12:**
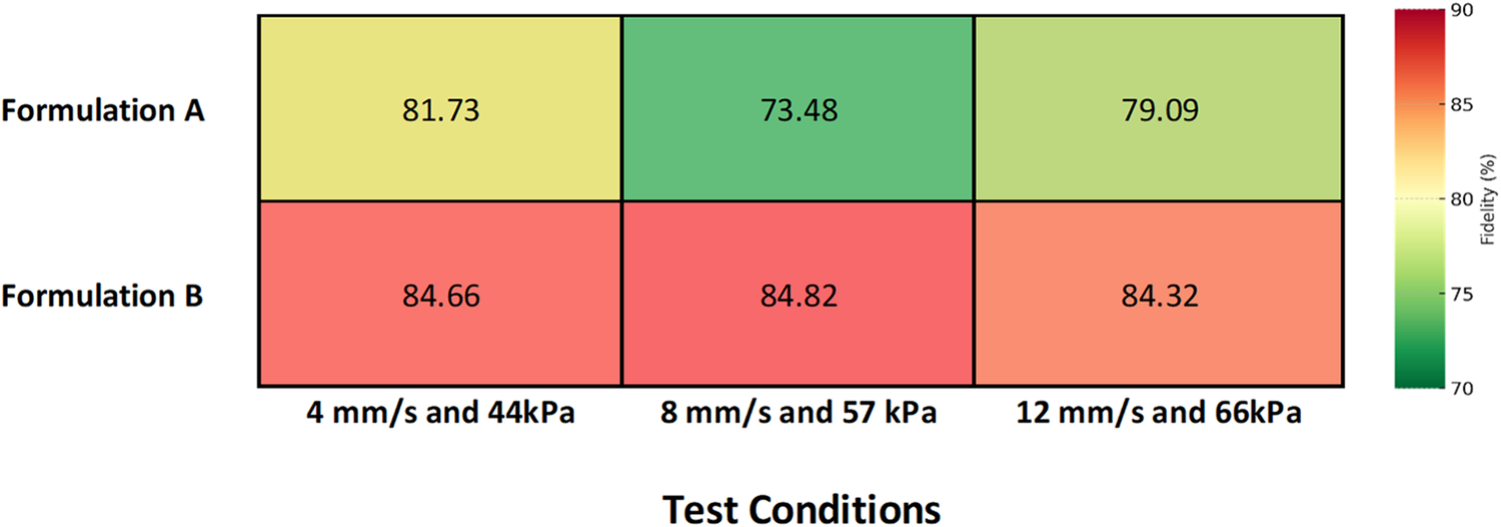
Combined printability map showing the mean lattice fidelity (A_real/A_theoretical × 100) for Formulations A and B under different combinations of extrusion pressure and printing speed. The color scale represents the percentage of dimensional fidelity, where higher values (red tones) indicate better structural accuracy.

Formulation B exhibited a higher average geometric fidelity across all three tested conditions (84.66, 84.82, and 84.32% for B.1, B.2, and B.3, respectively), producing more continuous, uniform, and well-defined filaments. In contrast, Formulation A showed greater variability and lower average fidelity (81.73, 73.48, and 79.09%), with a tendency toward local deformations and instances of overextrusion.

Taken together, these results confirm that Formulation B performs better during printing, maintaining complex structures with higher dimensional precision even under increased speed or extrusion pressure, making it a more robust option for bioprinting applications requiring reproducibility and structural stability.

Figure 13 presents the distribution of shape fidelity values measured for each printed lattice. Formulation A showed greater variability between grids, particularly at intermediate conditions (A2), reflecting lower reproducibility and print stability. In contrast, Formulation B achieved consistently higher median fidelity and narrower dispersion, confirming its superior structural accuracy and reproducibility under the tested printing parameters. These observations agree with the optimal printing window identified in the printability map (Figure 12).

**Figure 13:**
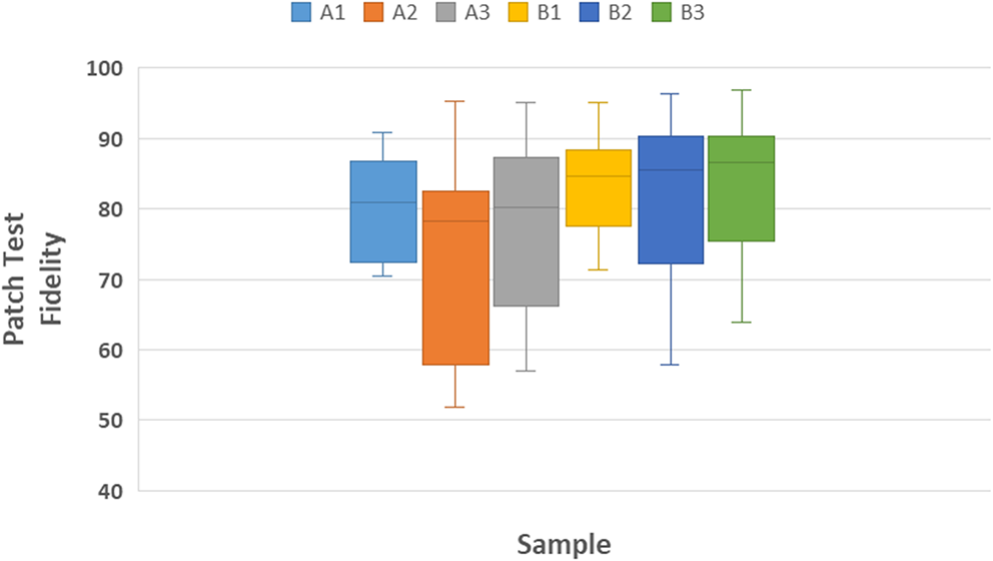
Box plots representing the distribution of shape Fidelity. obtained from the printing grid test for each printed lattice (A1–B3). Each box summarizes the variability among independently printed grids under the same formulation and printing condition.

### 3.4 Comparative Analysis of the Formulations

The development of bioinks based on natural polysaccharides represents a promising strategy for advancing toward more sustainable, safer, and accessible bioprinting. In this study, two formulations (A and B) were comparatively evaluated, designed using optimal concentrations of κ-carrageenan (KC), tragacanth gum (TG), and konjac glucomannan (KG), selected based on their rheological behavior and cytotoxicity.

From a mechanical standpoint, rheological tests revealed significant differences between the two formulations. Formulation B, containing 2% KG, displayed dominant solid-elastic behavior up to 34.5 °C, followed by a gradual loss of stiffness. This profile indicates a robust structural network capable of maintaining shape during and after the printing process. In contrast, Formulation A (1.5% KG) showed predominantly viscous behavior up to 37 °C, with *G*″ > *G*′, suggesting a less cohesive network, more sensitive to temperature, and potentially less stable after deposition.

In terms of biocompatibility, both formulations exceeded the 70% cell viability threshold established by ISO 10993–5. However, Formulation A showed slightly higher values (86.51% at 24 h and 82.14% at 48 h) compared to Formulation B (72.68 and 72.83%, respectively). This difference could be partly attributed to the lower total solute concentration in Formulation A (7.5% hydrogel versus 8% in Formulation B), which reduces local osmotic pressure and limits unfavorable interactions between the gel and the cells, thus promoting a more cell-friendly environment. Moreover, although the KG concentration difference is only 0.5%, the impact on viscoelasticity is notable and may also contribute to the variation in cytotoxicity.

The printability of both formulations was validated through bridging and patch tests. Formulation B demonstrated greater self-supporting ability, maintaining spans of up to 6 mm without apparent collapse, and consistently showed higher geometric fidelity under various printing conditions (84–85%). In contrast, Formulation A showed premature collapse in the bridging test and greater variability in printing fidelity, especially at higher extrusion speeds.

As a limitation, this study focused on short-term evaluations (48 h), without including long-term cell viability analysis, cell adhesion tests, or postprinting mechanical evaluations—factors that will be addressed in future research.

In summary, these results highlight the inherent trade-off between structural rigidity and biocompatibility that characterizes many natural bioink systems. While Formulation B emerges as a more suitable option for printing complex, self-supporting 3D structures, Formulation A may be more favorable in contexts where prolonged cell viability is a priority, such as long-term cell models or highly sensitive cultures.

## 4 Conclusions

This study reports the design, formulation, and characterization of two bioinks derived from natural polysaccharides, κ-carrageenan (KC), tragacanth gum (TG), and konjac glucomannan (KG); using an integrated evaluation framework that included rheological testing, cytocompatibility assays, and printability analysis. Based on the comparative results, the following conclusions were established:

1. The optimal concentrations balancing mechanical strength and biocompatibility were identified as 2% KC, 4% TG, and 1.5–2% KG, depending on the formulation and its intended application.
2. Both formulations (A and B) demonstrated rheological properties suitable for extrusion-based bioprinting and surpassed the ISO 10993–5 threshold for cell viability, confirming their safety as bioink candidates.
3. Formulation B (2% KG) exhibited a stronger structural network and higher geometric fidelity during printing, making it more suitable for complex structures requiring high dimensional accuracy.
4. Formulation A (1.5% KG) provided enhanced short-term cell viability, positioning it as the preferred option for applications prioritizing biocompatibility, despite lower structural robustness.
5. Both formulations present opportunities for further optimization, particularly through the incorporation of bioactive molecules (e.g., growth factors, peptides) to improve biological performance without significantly compromising mechanical integrity.
6. The findings validate the potential of food-grade polysaccharide blends as versatile, modular, and adaptable bioink platforms, while the applied methodology, combining rheology, cytotoxicity, and printability, offers a transferable framework for evaluating other polymeric systems.
7. These bioinks hold promise for applications such as bioprinted tissue patches, personalized soft tissue models, and controlled release platforms, contributing to the advancement of sustainable, safe, and accessible biomedical solutions.

Finally, although the HEK293T cell model is not tissue-specific, it provides a robust and reproducible platform for preliminary biocompatibility assessment. This strategy allowed the present study to focus on the physicochemical properties and printing fidelity of the developed bioink. Future work will build on these findings by incorporating tissue-specific cell lines to evaluate the biological performance of the KC–TG–KG system in more physiologically relevant contexts.

## Data Availability

The authors confirm that the data supporting the findings of this study are available within the article.
